# Long noncoding RNA LINC00857 promotes pancreatic cancer proliferation and metastasis by regulating the miR-130b/RHOA axis

**DOI:** 10.1038/s41420-022-01008-2

**Published:** 2022-04-13

**Authors:** Peng Chen, Zhirui Zeng, Jie Wang, Wenpeng Cao, Chunzhuo Song, Shan Lei, Yichuan Li, Zhangxia Ren

**Affiliations:** 1 Department of General Surgery, Guang’an People’s Hospital, Guang’an, Sichuan China; 2 Basic Medical College of Guizhou Medical University, Guiyang, Guizhou China; 3 Department of Hepatobiliary Surgery, Renmin Hospital of Wuhan University, Wuhan, Hubei China; 4 Department of Hepatobiliary Surgery, The Second affiliated Hospital of Army Medical University, Chongqing, China

**Keywords:** Metastasis, Cancer microenvironment

## Abstract

Dysregulation of long noncoding RNAs (lncRNAs) is involved in the pathogenesis and progression of pancreatic cancer (PC). In the current study, we investigated the role and molecular mechanism of LINC00857 in PC. The expression of LINC00857 in PC was analyzed by bioinformatics analysis and qRT-PCR, and the relationship between LINC00857 expression and clinical characteristics of patients of PC was analyzed by Fisher’s exact test. Gain- and loss-of-function assays were performed to determine the biological function of LINC00857 in PC. The relationship between LINC00857, miR-130b, and RHOA were determined by RNA pull-down assay, luciferase assay, and qRT-PCR. Our results demonstrated that LINC00857 expression was elevated in PC, and high expression of LINC00857 was positively associated with tumor diameter, T stage, and lymph node metastasis. LINC00857 promoted the proliferation and mobility of PC cells in vitro and in vivo. Mechanistically, LINC00857 acts as a sponge for miR-130b and decreases its expression. miR-130b exhibits tumor suppressor functions in PC, and RHOA was identified as the key target gene of miR-130b. The functions induced by LINC00857 in PC cells were dependent on the miR-130b/RHOA axis. In conclusion, the current study indicated that LINC00857 promotes PC tumorigenesis and metastasis by modulating the miR-130b/RHOA axis, implying that LINC00857 might be a new therapeutic target for PC.

## Introduction

Pancreatic cancer (PC) is a malignant tumor of the digestive system, with a 5-year survival rate of only 9%. The incidence of PC has steadily increased worldwide over the past three decades [1]. Surgical treatment is the most effective treatment for pancreatic cancer. However, because of the lack of clinical symptoms in the early stage, most patients lose the opportunity for surgical treatment at diagnosis, and only 20% of the patients diagnosed with PC are candidates for initial resection [2, 3]. In addition, adjuvant treatments have limited effects [4] and are associated with serious side effects including liver and kidney damage. Therefore, better understanding of the underlying molecular mechanisms of PC for the identification of novel biomarkers for diagnosis and treatment targets is needed.

Long noncoding RNAs (lncRNAs) are RNA molecules >200 nucleotides in length that lack coding function [5]. Many studies have shown the aberrant expression of lncRNAs participated in a series of biological processes, including cell cycle regulation, apoptosis, metastasis, aging [6], and chemotherapy resistance [7]. Moreover, lncRNAs affect the progression of cancer by regulating downstream targets and related signal pathways and can act as oncogenes or tumor suppressors. One study reported that lncRNA PMSB8-AS1 is highly expressed in PC and acts as an oncogene to promote PC cell proliferation and mobility by regulating miR-382-3p/STAT1 [8]. Shahabi et al. revealed that LINC00261 functions as a tumor suppressor to inhibit PC cell proliferation via inducing DNA damage [9]. Generally, the abnormal expression of lncRNAs involving in the occurrence and progression of tumor via the competing endogenous RNA (ceRNA) regulating network [10], post-transcriptional regulation, metabolic, reprogramming [11], the ceRNA regulation network is currently most frequently reported molecule mechanism to explain the function in tumor [12], LncRNA can increase or decrease the expression of downstream target genes by sponging target miRNAs and play a role in tumorigenesis and development [13].

RHO family proteins, including RHOA, RHOB, and RHOC, are members of the Ras superfamily. RHO proteins are guanosine triphosphate (GTP) binding proteins with a relative molecular weight of ~20–25 kD. RHO proteins have GTP enzyme activity and thus are commonly called RHO GTP enzymes [14, 15]. RHO GTP enzymes regulate cytoskeleton reorganization [16]. RHO family proteins are upregulated in tumors and can mediate the malignant biological behavior of cancer. For example, Zhou et al. reported that under hypoxia condition, Hif-3α promotes PC cell invasion and metastasis by regulating the RHOC-ROCK1 signaling pathway [17]. Wang et al. demonstrated that miR-106b-5p promotes the progression of breast cancer by increasing activation of the RHO/ROCK1 pathway [18].

In the present research, we aimed to identify the role and molecular mechanism of LINC00857 in PC. We found that LINC00857 was highly expressed in PC and positively associated with adverse clinical characteristics. Our data demonstrate that LINC00857 promotes PC cell proliferation and motility via the miR-130b/RHOA axis. These results indicate that LINC00857 may be a potential target for PC diagnosis and therapy.

## Results

### LINC00857 expression was elevated in PC tissues and related to a poor prognosis

**Fig. 1:**
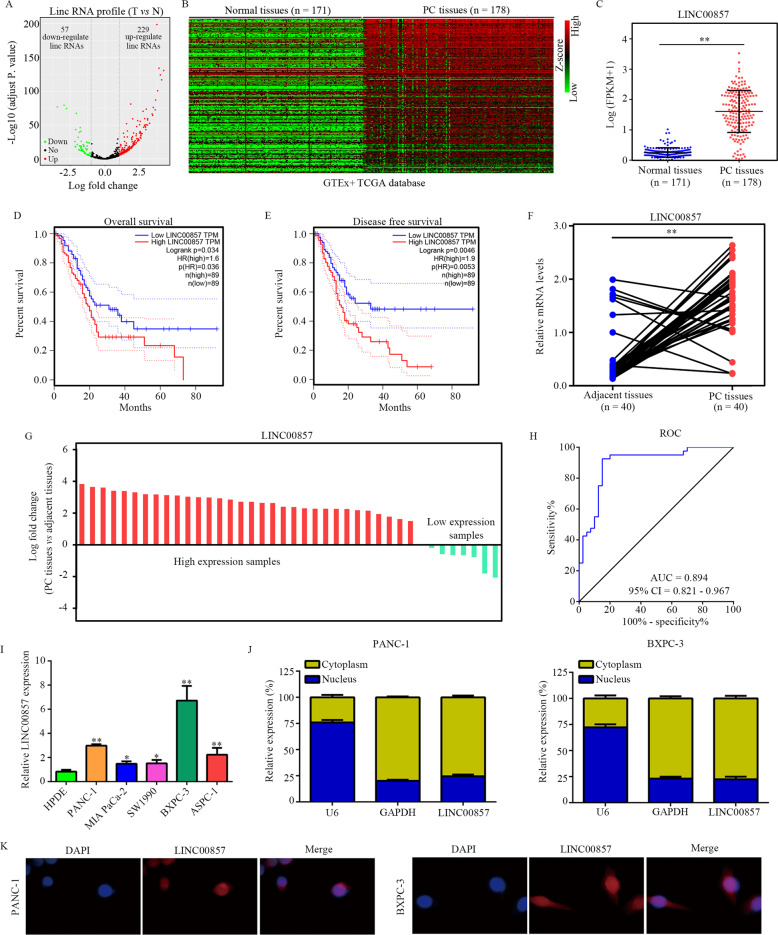
LINC00857 was upregulated in PC tissues and associated with a poor prognosis. **A** Volcano showed the differently expressed lncRNAs between PC tissues and normal tissues in TCGA and GTEx database. **B** Heatmap showed the differently expressed lncRNAs between PC tissues and normal tissues. **C** Expression of LINC00857 in PC tissues and normal tissues in TCGA and GTEx database is shown. **D** The overall survival rate of PC patients with high and low LINC00857 expression based on TCGA database. **E** The disease-free survival rate of PC patients with high and low LINC00857 expression based on TCGA database. **F**, **G** The expression of LINC00857 in PC tissues and corresponding adjacent tissues from our research group was determined. **H** ROC analysis demonstrated that LINC00857 had a high diagnostic value to distinguish PC tissues and adjacent normal tissues. **I** qRT-PCR showed the LINC00857 expression in PC cell lines (PANC-1, MIA PACA-2, SW1990, BXPC-3, and ASPC-1) and normal pancreatic epithelial cells (HPDE). **J** the relative expression of LINC00857 in nucleus and cytoplasm. **K** RNA-FISH showed the localization of LINC00857 in PC cells. *P < 0.05, **P < 0.01.

Through analyzing the gene expression data of PC tissues (*N* = 178) and normal pancreatic tissues (*N* = 171) in TCGA and GTEx databases, we identified 57 downregulated lncRNAs and 229 upregulated lncRNAs in PC (Fig. 1A, B). Among these differentially expressed lncRNAs, LINC00857 was selected for analysis because its prognostic value. LINC00857 expression was significantly increased in PC tissues compared with normal pancreatic tissues (Fig. 1C). PC patients with high LINC00857 expression had a lower overall survival rate (Fig. 1D) and disease-free survival rate (Fig. 1E). qRT-PCR analysis of LINC00857 expression in PC and adjacent normal tissues showed that LINC00857 was elevated in PC tissues (Fig. 1F, G), and LINC00857 had high diagnostic value to distinguish PC tissues and adjacent tissues (Fig. 1H). Expression of LINC00857 was significantly associated with tumor diameter, pathological T, and lymph node metastasis (Supplementary Table 2). LINC00857 expression in PC cell lines including PANC-1, MIA PaCa-2, SW1990, BXPC-3, and ASPC-1 cell lines was elevated compared with the expression in HPDE cells (Fig. 1I). LINC00857 was mostly localized in the cytoplasm of PC cells (Fig. 1J, K).

### LINC00857 promotes PC cell proliferation, migration, and invasion in vitro

**Fig. 2:**
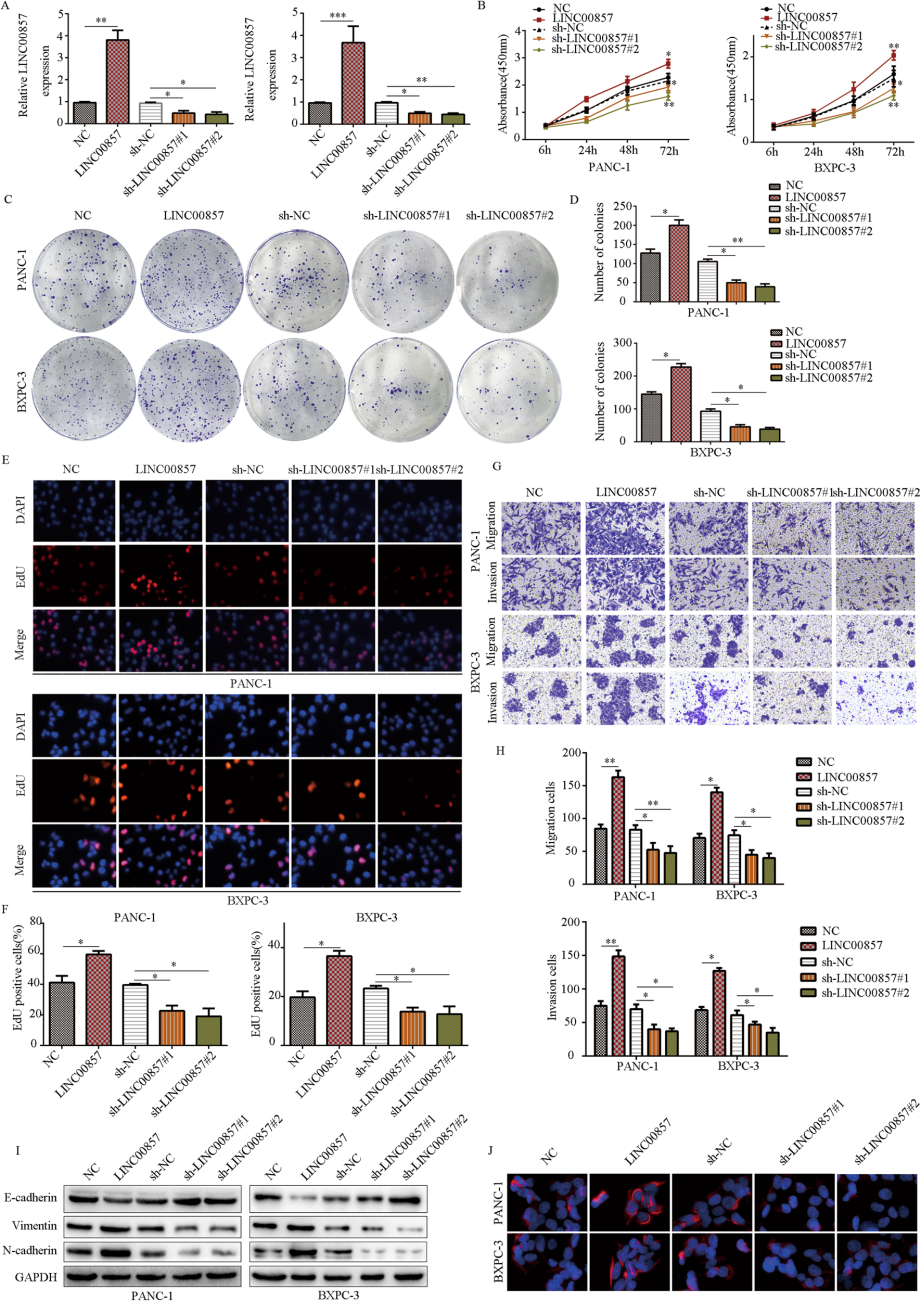
LINC00857 promotes PC cell proliferation, migration, and invasion in vitro. **A** qRT-PCR was used to examine the lentivirus transfection efficiency. **B** CCK-8 assays were performed to detect the effects of LINC00857 on PC cell proliferation. **C**, **D** Colony-formation assays were performed to detect the effects of LINC00857 on PC cell colony formation. **E**, **F** EDU-positive rate in PC cells with LINC00857 overexpression and knockdown was determined. **G**, **H** The transwell assay was conducted to test the effects of LINC00857 on PC cell migration and invasion. **I** The protein expression of E-cadherin, N-cadherin and vimentin in PC cells with LINC00857 overexpression and knockdown was determined by western blot. **J** The protein expression of vimentin in PC cells with LINC00857 overexpression and knockdown was determined by immunofluorescence. *P < 0.05, **P < 0.01.

To evaluate the potential biological role of LINC00857 in PC cells in vitro, we used lentivirus for LINC00857 overexpression or downregulation in PANC-1 and BXPC-3 cells (Fig. 2A). CCK-8 and clone formation assays demonstrated that upregulation of LINC00857 significantly promoted the proliferation and clone formation activity of PC cells, while downregulation of LINC00857 decreased proliferation and clone formation (Fig. 2B–D). EDU assays demonstrated that PC cells with high LINC00857 expression had a higher EDU-positive rate, while those with downregulated LINC00857 expression had a lower EDU-positive rate (Fig. 2E, F). Next, we conducted Transwell assays to evaluate the effect of LINC00857 on the migration and invasion of PC cells. Compared with the control group, the LINC00857 overexpression group showed significantly increased invasion and migration, while the LINC00857 downregulation group showed decreased PC cell invasion and migration (Fig. 2G, H). Furthermore, increasing LINC00857 elevated E-cadherin expression and reduced N-cadherin and Vimentin expression, while LINC00857 downregulation induced the opposite effects (Fig. 2I). Immunofluorescence further demonstrated that overexpression of LINC00857 increased Vimentin expression, while LINC00857 knockdown reduced Vimentin expression (Fig. 2J).

### LINC00857 promotes PC cells proliferation and metastasis in vivo

**Fig. 3:**
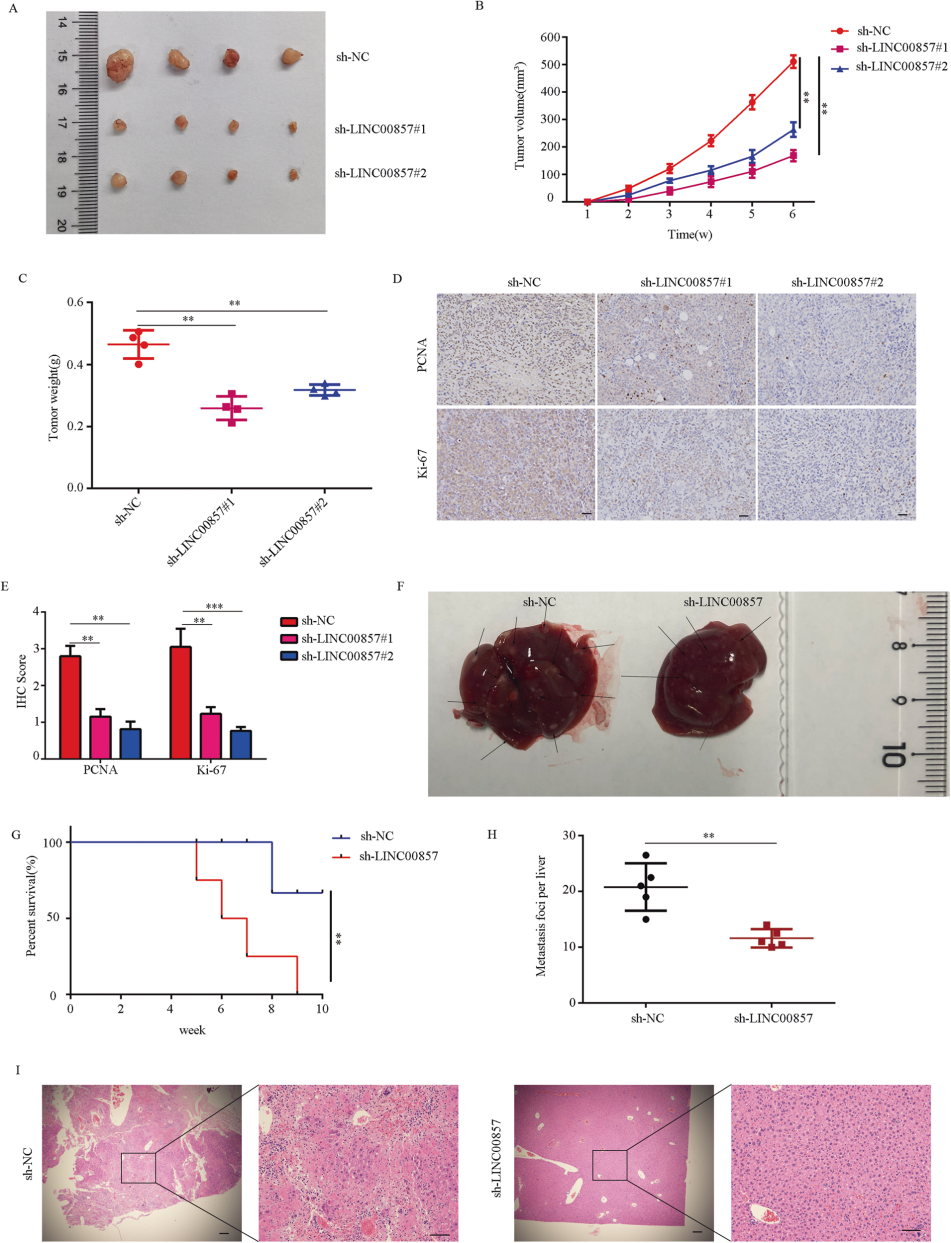
LINC00857 promotes PC cells proliferation, migration, and invasion in vivo. **A** Tumor photographs subcutaneous xenografts in sh-NC, sh-LINC00857#1, and sh-LINC00857#2 groups (*n* = 4 for each group). **B** Tumor volume change curve of sh-NC, sh-LINC00857#1, and sh-LINC00857#2. **C** Tumor weight was weighed in the mice in sh-NC, sh-LINC00857#1, and sh-LINC00857#2 group. **D**, **E** IHC staining score and representative images of Ki-67 and PCNA in the tumor tissues of sh-NC, sh-LINC00857#1, and sh-LINC00857#2. **F** Liver metastasis photographs indicated metastasis loci. **G** Survival carve showed the prognosis of mice in sh-NC and sh-LINC00857 group. **H** The number of liver micro-metastases was counted. **I** Serial section of the whole liver was H&E stained. **P < 0.01.

To further determine the biological effects of LINC00857 in vivo, PANC-1 cells stably transfected with the lentivirus used above were injected into nude mice to construct subcutaneous tumor models and liver metastasis models. In the subcutaneous tumor model, the tumor volume (Fig. 3A, B) and tumor weight (Fig. 3C) in the LINC00857 knockdown groups (sh-LINC00857#1 and sh-LINC00857#2) were significantly reduced compared with those of the control group (3A–B). Immunohistochemistry of tumor specimens revealed lower Ki-67 and PCNA expression in the LINC00857 knockdown group compared with the high LINC00857 expression (Fig. 3D, E).

In the liver metastasis model, the number of liver metastases in LINC00857 knockdown group was significantly decreased compared with the number in the high LINC00857 expression group (Fig. 3F). Overall survival was significantly longer in mice in the high LINC00857 expression group (Fig. 3G). Statistical analysis of tumor liver metastasis number and HE stain further confirms this result (Fig. 3H, I). Together, these results indicated that LINC00857 promotes PC cell proliferation and metastasis in vivo.

### LINC00857 sponges miR-130b and is negatively associated with miR-130b expression

**Fig. 4:**
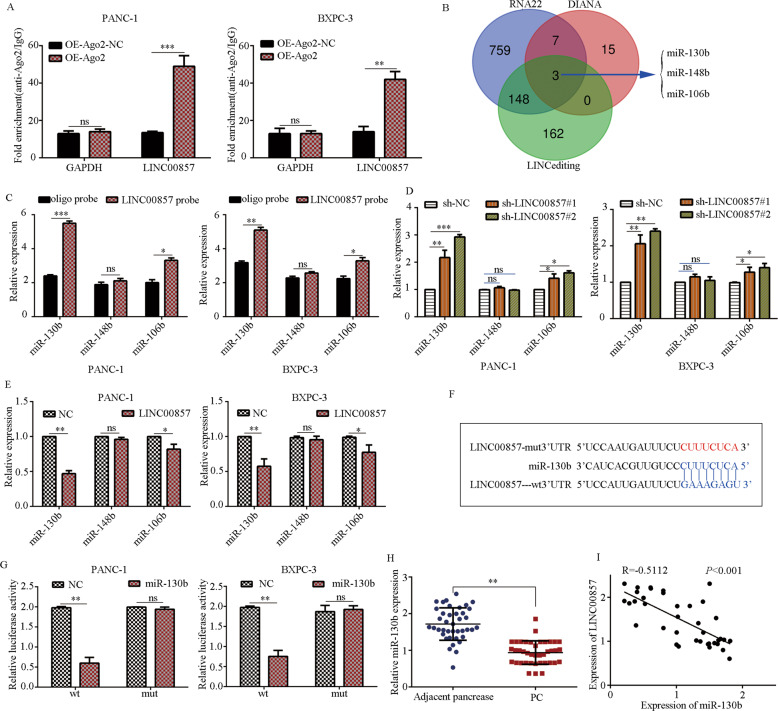
LINC00857 sponges with miR-130b. **A** A RIP assay was performed to assess the LINC00857 level in PC cells transfected with Ago2 overexpression vectors or control vectors. **B** Schematic illustration showing the overlapping target miRNAs of LINC00857 predicted by DIANA, LINC editing, and RNA22. **C** An RNA pull-down assay was performed to determine the binding between LINC00857 and target miRNAs. **D**, **E** The expression of miR-130b, miR-148b, and miR-106b in PC cells with LINC00857 overexpression and knockdown. **F** The binding site between LINC00857 and miR-130b. **G** Luciferase activity was analyzed in PC cells after transfected LINC00857 wt or LINC00857 mut and miR-130b mimics or NC mimics. **H** The miR-130b expression was evaluated in PC tissues and adjacent tissues. **I** The co-expression relationship between miR-130b and LINC00857 was determined by qRT-PCR. *P < 0.05, **P < 0.01.

Given that LINC00857 was mostly localized in the cellular cytoplasm, we speculated that LINC0857 may perform its biological function via a ceRNA mechanism. We conducted a RIP assay using anti-Ago2 antibody in PANC-1 and BXPC-3 cells transfected with the Ago2-overexpression plasmid or NC vector. More endogenous LINC00857 was immunoprecipitated in cells transfected with the Ago2 overexpression plasmid compared with cells transfected with the NC vector (Fig. 4A). This evidence further suggests that LINC00857 may perform functions through the ceRNA mechanism.

Next, three candidates target miRNAs that contained miR-130b, miR-148b, and miR-106b were predicted by bioinformatics methods (RNA22, DIANA, and LINC editing) (Fig. 4B). We conducted pull-down assays using a biotinylated probe specific for LINC00857. The results showed that miR-130b was pulled down by LINC00857 probes in both PANC-1 and BXPC-3 cells (Fig. 4C). We next examined the expression levels of miR-130b, miR-148b, and miR-106b in cells in response to modulated expression of LINC00857. miR-130b was increased in PANC-1 and BXPC-3 cells with LINC00857 downregulation (Fig. 4D) and decreased in PANC-1 and BXPC-3 cells with LINC00857 overexpression (Fig. 4E).

We identified binding sites in LINC00857 and miR-130b (Fig. 4F). We then conducted a dual-luciferase reporter assay. Co-transfection with the miR-130b mimic inhibited the luciferase activity of the WT LINC00857 reporter vector but not the MUT reporter vector (Fig. 4G). qRT-PCR revealed that miR-130b expression was decreased in PC tissues (Fig. 4H) and miR-130b expression was inversely associated with LINC00857 expression (Fig. 4I).

### Inhibition of miR-130b reversed the suppressive effects of LINC00857 knockdown on PC cell proliferation and mobility

**Fig. 5:**
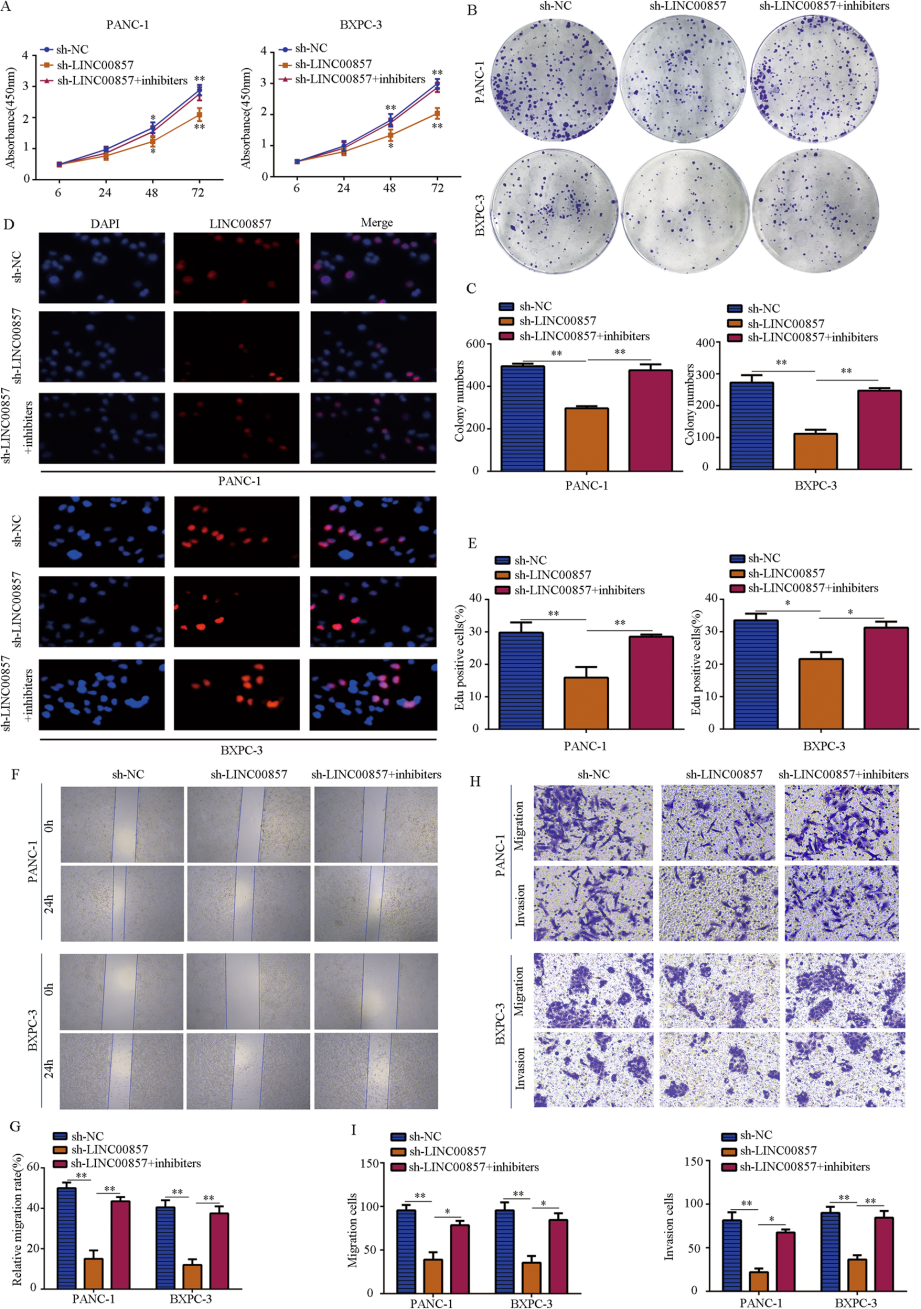
miR-130b inhibition reversed the effects of LINC00857 knockdown on PC cell proliferation and mobility. PC cells were transfected with sh-NC, sh-LINC00857, and sh-LINC00857 plus miR-130b inhibitors, respectively. **A** CCK-8 assay was used to detect the proliferation rate in each group. **B**, **C** Colony-formation assay was used to detect the colony formation in each group. **D**, **E** EDU assay was used to detect the EDU-positive rate in each group. **F**, **G** Wound-healing assay was used to detect the migration in each group. **H**, **I** Transwell assay was used to detect the invasion in each group. *P < 0.05, **P < 0.01.

Since the expression of LINC00857 is significantly negatively correlated with miR-130b in PC tissues, functional rescue experiments were performed. CCK-8 assay (Fig. 5A) and colony-formation assay (Fig. 5B, C) demonstrated that inhibition of miR-130b reversed the suppressive effects of LINC00857 knockdown on PANC-1 and BXPC-3 cell proliferation and colony formation. While LINC00857 knockdown dramatically decreased the number of EdU-positive cells in PANC-1 and BXPC-3, inhibition of miR-130b partially reversed these effects (Fig. 5D, E). Wound-healing assay (Fig. 5F, G) and Transwell assays (Fig. 5H, I) revealed that while PC cell migration and invasion were decreased in the cells in the sh-LINC00857 group, these effects were reversed upon inhibition of miR-130b.

### RHOA is a target of miR-130b

**Fig. 6:**
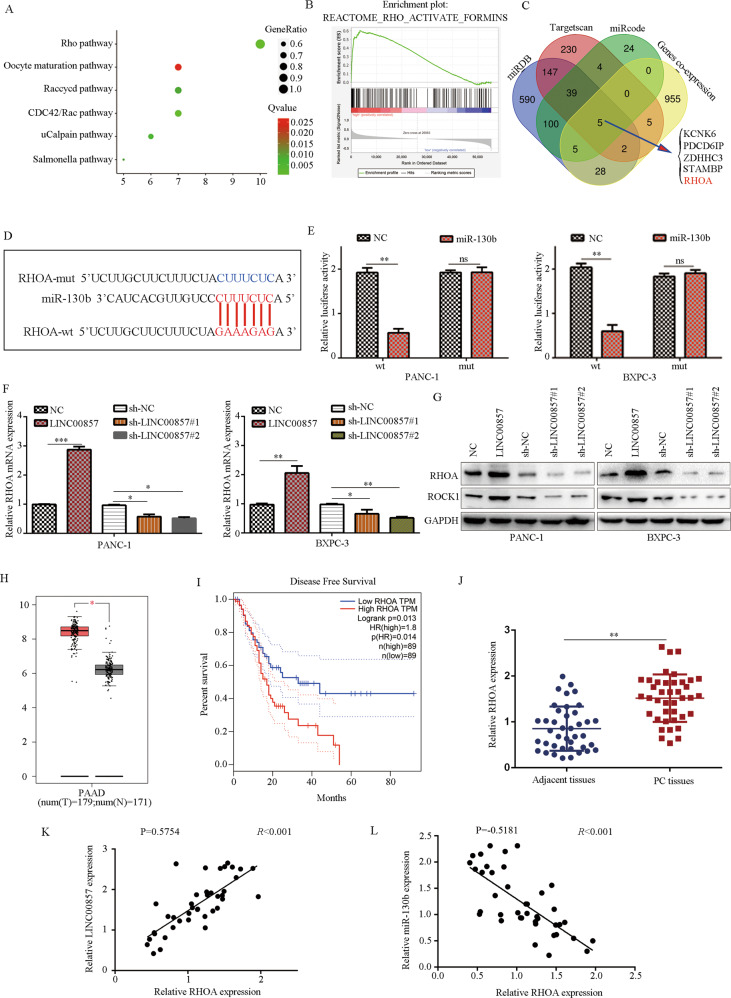
LINC00857 positively regulates RHOA expression in PC cells by sponging miR-130b. **A** Pathway analysis showed that pathways which LINC00857 co-expressed genes enriched in. **B** GSEA analysis demonstrated that LINC00857 significantly and positively associated with RHO pathway activation**. C** Schematic illustration showing the overlapping target genes of miR-130b predicted by miRDB, TargetScan, miRcode and gene co-expression of LINC00857. **D** The binding site between miR-130b and RHOA is shown. **E** Dual-luciferase activity was analyzed in PC cells after transfected RHOA wt or RHOA mut and miR-130b mimics or NC mimics. **F** RHOA mRNA expression levels in PC cells with LINC00857 overexpression and knockdown were evaluated by qRT-PCR. **G** Western blot was performed to determine RHOA and ROCK protein expression in PC cell with LINC00857 overexpression and knockdown. **H**, **I** Based on the TCGA database, it was demonstrated that RHOA was upregulated in PC tissues, while PC patients with high RHOA expression had a low disease-free survival rate. **J** The mRNA level of RHOA in PC tissues and adjacent tissues is shown. **K** The co-expression relationship between LINC00857 and RHOA is shown. **L** The co-expression relationship between miR-130b and RHOA is shown. *P < 0.05, **P < 0.01.

To further determine the molecular mechanism of LINC00857 in PC, we obtained the co-expression genes in PC in TCGA database and performed pathway enrichment analysis. Results demonstrated that co-expression genes with PC were the most significantly enriched into the Rho pathway (Fig. 6A). GSEA analysis also demonstrated that LINC00857 had significant potential to promote activation of the RHO pathway (Fig. 6B). We then analyzed the target genes of miR-130b regulated by LINC00857, online tool miRDB, TargetScan and miRcode were used. Through intersection analysis between predicted target genes of miR-130b and co-expression genes of LINC00857, we identified five genes: KCNK6, PDCD6IP, ZDHHC3, STAMBP, and RHOA (Fig. 6C). RHOA is a key factor in the RHO pathway and therefore we focused on RHOA.

After obtaining the binding site between RHOA and miR-130b (Fig. 6D), we conducted a dual-luciferase reporter assay. Luciferase activity was significantly decreased in cells co-transfected with the WT RHOA plasmid and miR-130b mimic, but not in cells transfected with the MUT RHOA plasmid and miR-130b mimic (Fig. 6E). qRT-PCR demonstrated that overexpression of LINC00857 increased the expression of RHOA, while downregulation of LINC00857 reduced the expression of RHOA (Fig. 6F). LINC00857 overexpression increased the expression of RHOA and the downstream protein ROCK1 expression, while downregulated LINC00857 expression reduced the expression of RHOA and ROCK1 (Fig. 6G).

We found that RHOA expression was upregulated in PC tissues in comparison with normal tissues based on the results from TCGA and GTEx (Fig. 6H), and patients with high ROCK1 expression had a lower survival rate (Fig. 6I). RHOA expression was also higher in PC samples (Fig. 6J), and RHOA expression was positively associated with LINC00857 expression (Fig. 6K) and negatively associated with miR-130b expression (Fig. 6L).

### RHOA overexpression reversed the inhibitory effects of miR-130b on PC cell proliferation and motility

**Fig. 7:**
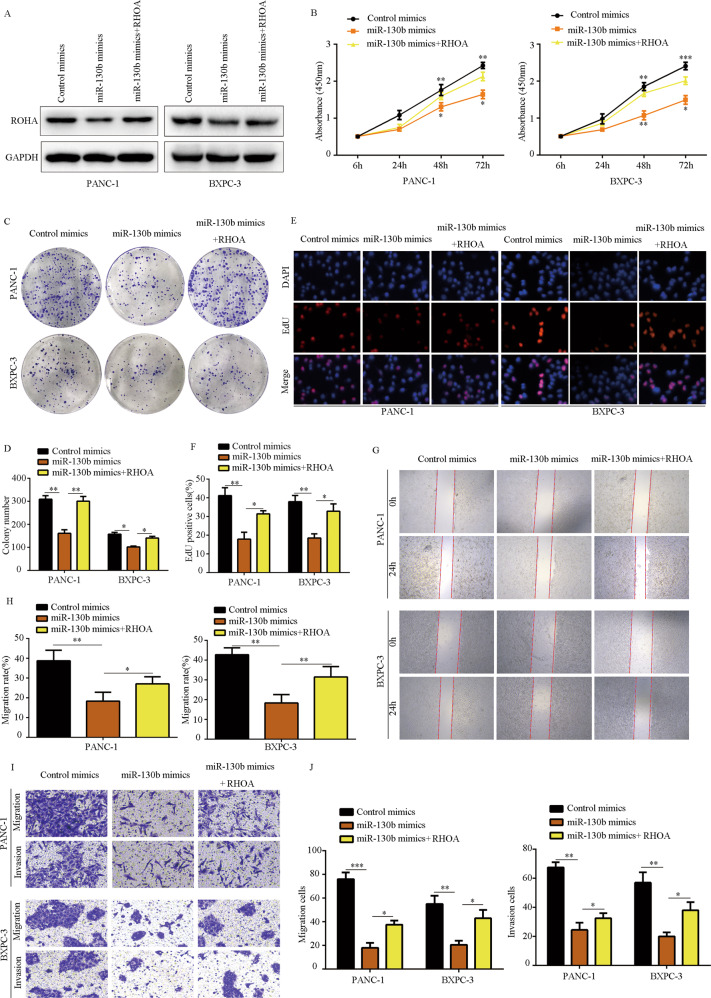
RHOA overexpression reversed the inhibitory effects of miR-130b on PC cell proliferation and mobility. PC cells were transfected with control mimic, miR-130b mimic, and miR-130b plus RHOA lentivirus, respectively. **A** Western blot was used to detect the expression of RHOA in each group. **B** CCK-8 assay was used to detect the proliferation rate in each group. **C**, **D** Colony-formation assay was used to detect the colony formation in each group. **E**, **F** EDU assay was used to detect the EDU-positive rate in each group. **G**, **H** Would-healing assay was used to detect the migration in each group. **I**, **J** Transwell assay was used to detect the invasion in each group. *P < 0.05, **P < 0.01.

We next examined whether RHOA was involved in the biological functions affected by miR-130b. Western blot showed that RHOA expression was significantly decreased in PC cells with miR-130b overexpression, and the expression of RHOA was partially increased in PC cells transfected with miR-130b mimics plus RHOA plasmid (Fig. 7A). CCK-8 assay (Fig. 7B) and colony-formation assay (Fig. 7C, D) demonstrated that RHOA overexpression significantly reversed the suppressive effects induced by miR-130b on PC cell proliferation and colony formation. RHOA overexpression reversed the decrease of EDU-positive rate in PC cells with miR-130b overexpression (Fig. 7E, F). Furthermore, while the migration and invasion of PC cells were reduced in cells with miR-130b overexpression, overexpression of RHOA reversed these effects (Fig. 7G–J). These results indicated that RHOA is involved in the biological functions regulated by miR-130b in PC cells.

### The effects of LINC00857 in PC cells were miR-130b/RHOA axis-dependent

**Fig. 8:**
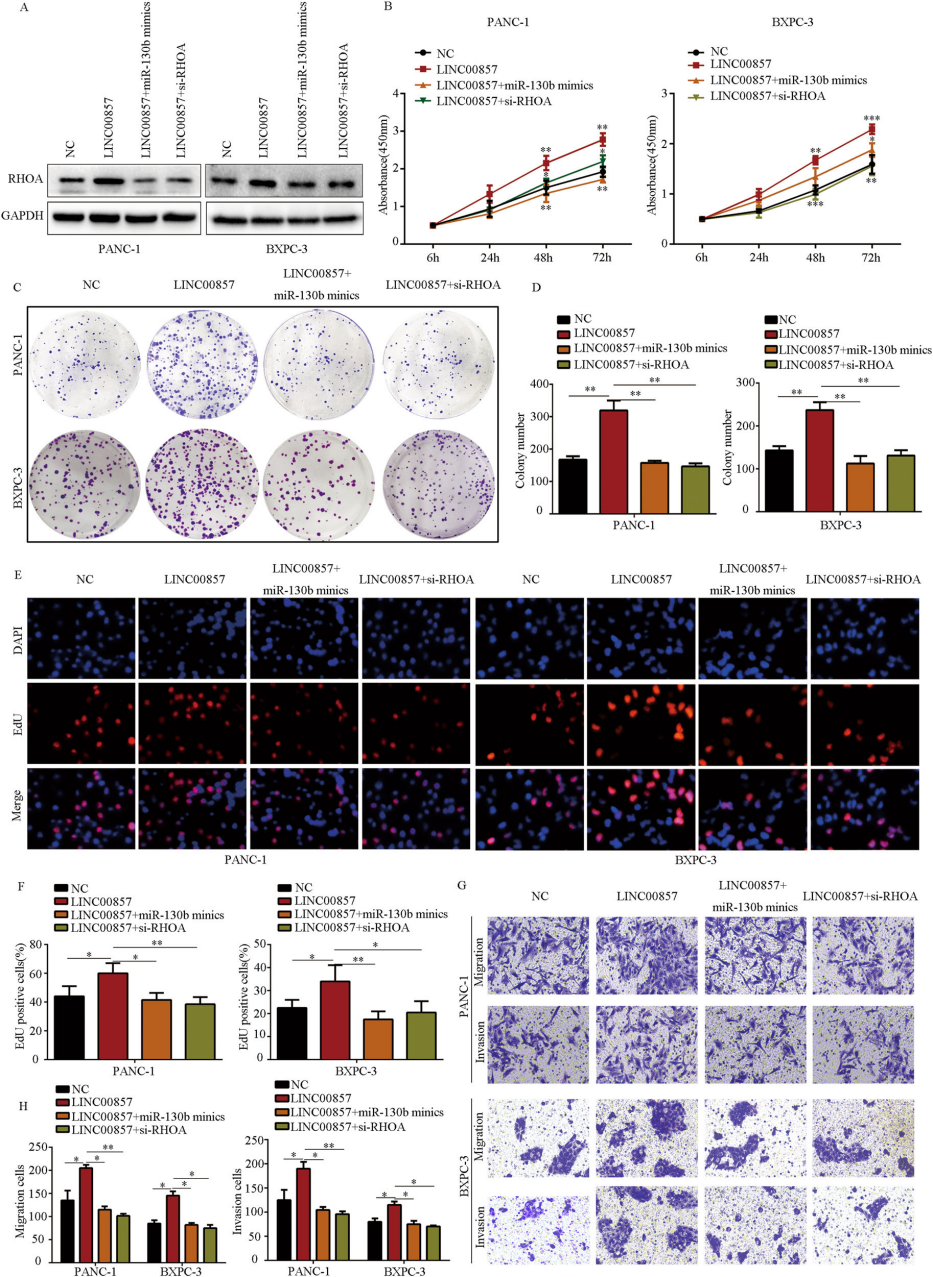
The effects of LINC00857 on PC cells were miR-130b/RHOA axis depend. PC cells were transfected with NC lentivirus, LINC00857 lentivirus, LINC00857 lentivirus plus miR-130b mimic, and LINC00857 lentivirus plus RHOA siRNA. **A** Western blot was used to detect the expression of RHOA in each group. **B** CCK-8 assay was used to detect the proliferation rate in each group. **C**, **D** Colony-formation assay was used to detect the colony formation in each group. **E**, **F** EDU assay was used to detect the EDU-positive rate in each group. **G**, **H** Transwell assay was used to detect the invasion in each group. *P < 0.05, **P < 0.01.

We performed rescue experiments to determine whether the effects of LINC00857 on PC were miR-130b/RHOA axis-dependent. Overexpression of miR-130b and RHOA inhibition reversed the stimulatory effects of LINC00857 on RHOA expression in PC cells (Fig. 8A). miR-130b overexpression and RHOA inhibition both significantly reduced the stimulating effects of LINC00857 on PC cell proliferation and colony formation (Fig. 8B–D). LINC00857 overexpression increased the EDU-positive rate in PC cells, while miR-130b overexpression and RHOA inhibition both significantly abolished these effects (Fig. 8E, F). Transwell assays demonstrated that while LINC00857 overexpression increased motility in PC cells, miR-130b overexpression and RHOA inhibition both significantly abolished these effects (Fig. 8G, H). These results indicated that the effects of LINC00857 on PC cells are miR-130b/RHOA axis-dependent (Supplementary Fig. S1).

## Discussion

With a high metastasis rate in early-stage, effective treatment of PC is a clinical challenge [19]. Although surgical resection combined with chemotherapy is an effective treatment for PC, only a small number of patients are eligible for surgical resection [20]. The lack of accurate biomarkers for early diagnosis further hinders the clinical treatment rates. Therefore, it is critical to explore the mechanism of occurrence and development of pancreatic cancer. Research has revealed the dysregulation of lncRNAs in cancer and the role of lncRNAs in cancer chemoresistance [21], proliferation, distant metastasis, autophagy, and glucose metabolism [22] in many human malignant tumors such as lung cancer [23], ovarian cancer [22]; esophageal adenocarcinoma [24], and gastric cancer [25]. However, understanding of the role of lncRNAs in PC has been limited.

In this study, we demonstrated that LINC00857 is involved in the progression of PC. Several studies reported that LINC00857 participates in the development of other malignant tumors. Lin et al. demonstrated that LINC00857 was upregulated in ovarian cancer and promotes ovarian cancer progression and glycolysis by regulating the Hippo signaling pathway [22]. LINC00857 is upregulated and acts as an oncogene in colorectal cancer to promote colorectal cancer proliferation, migration and invasion by interacting with YTHDC1 and stabilizing SLC7A5 [26]. Herein, we confirmed that LINC00857 is upregulated in PC using bioinformatics analysis based on TCGA database and revealed high expression of LINC00857 in PC tissues and several cell lines using RT-qPCR. LINC00857 was mostly localized in the cell cytoplasm. Analysis of the clinicopathological characteristics of 40 PC patients revealed that high LINC00857 level predicted lower survival rate, more advanced T stage, tumor node metastasis, and larger tumor size. Functional assays demonstrated that LINC00857 promotes PC cell proliferation and metastasis both in vitro and in vivo. These findings indicate the role of LINC00857 in the progression of PC.

MicroRNAs (miRNAs) are endogenous noncoding RNAs ~20–25 nucleotides in length with regulatory functions [27]. LncRNAs can function as a competitive endogenous RNA to sponge miRNA to block the binding of miRNA to downstream target mRNA, thereby resulting in the reduction or degradation of the downstream targets [28, 29]. A previous study showed that LINC00857 serves as an ceRNA that positively regulates CBX3 by negatively regulating miRNA370 expression, which promoted diffuse large B-cell lymphoma proliferation and lymphomagenesis [30]. We confirmed that LINC00857 binds the Ago2 protein, suggesting that LINC00857 may function through interacting with miRNAs. Candidate downstream miRNAs including miR-130b, miR-148b, and miR-106b were predicted through RNA22, DIANA, and LINCeding databases, and we found that only miR-130b exhibited a high binding capacity for LINC00857. qRT-PCR results further confirmed miR-130b was downregulated upon LINC00857 expression. miR-130b was previously reported to be involved in the pathological occurrence of multiple cancers [31]. We found that miR-130b expression was markedly reduced in PC tissues and its expression was negatively related to LINC00857 expression. Cell function assays further indicated that miR-130b inhibitors reversed the suppressive functions of LINC00857 knockdown on PC cell proliferation and metastasis.

RHOA is a Ras-related small GTP binding protein that plays a key role in a series of biological functions including cytoskeletal rearrangement, reactive oxygen species production, and regulation of cell morphology, cell movement, and transcription [32]. Yu et al. reported that CXCL12/CXCR4 positively regulates RhoA expression, further promoting inflammation-driven colorectal cancer progression by sponging miR-133a-3p [33]. In this study, from our TCGA database analysis results, we focused on the RHO pathway and found that RHOA is a target gene of miR-130b using bioinformatic analysis. Western blot and qRT-PCR results showed that RHOA expression was positively correlated with LINC00857 expression. In addition, RHOA expression was upregulated in PC and negatively correlated with disease-free survival of PC patients. Further functional rescue experiments showed that reduced RHOA expression partially reversed the effects of miR-130b overexpression on PC cells. Moreover, RHOA depletion or miR-130b overexpression inhibited the effects of LINC00857 on proliferation and metastasis of PC cells.

In conclusion, our research confirmed that LINC00857 promotes the progression of PC via the miR-130b/RHOA axis and may become a new therapeutic target in PC.

## Materials and methods

### Bioinformatics analysis

The lncRNA expression data of PC patients was obtained from The Cancer Genome Atlas (TCGA; https://portal.gdc.cancer.gov/). The lncRNA expression data of normal pancreas were downloaded from Genotype-Tissue Expression (GTEx; https://commonfund.nih.gov/GTEx/). All lncRNA expression data were merged and normalized via sva package. All data were converted as Log2 form and analyzed in R software (Version: 4.0.5) using edgeR package. LogFC>1 and adjusted *P* value <0.05 were set as a threshold for significance.

### Human PC tissue samples

Forty pairs of PC and adjacent tissues were obtained from surgical specimens from inpatients in the Affiliated Hospital of Guizhou Medical University from 2016 to 2020. The specimens were stored in liquid nitrogen. The Human Ethics Committee of Guizhou Medical University approved the collection and use of these samples.

### Cell culture and transfection

PC cell lines were purchased from American Type Culture Collection (ATCC). The human pancreatic ductal epithelial cell (HPDE), ASPC, and BXPC-3 cells were cultured in RPMI 1640 medium (Hyclone, USA) supplemented with 10% fetal bovine serum (Gibco, USA). PANC-1, MIA PaCa-2, and SW1990 cells were cultured in DMEM medium (Hyclone, USA) supplemented with 10% fetal bovine serum (Gibco, USA). All cells were cultured in 5% CO_2_ at 37 °C. The LINC00857 overexpression lentiviral vector (LINC00857), LINC00857 shRNA lentiviral vectors (sh-LINC00857#1 and LINC00857#2), the corresponding negative control lentiviral vectors (NC and sh-NC), miR-130b mimics, control mimic, miR-130b inhibitors, and control inhibitor were purchased from Ribobio (Guangzhou China). The RHOA overexpression lentiviral vector (RHOA), negative control lentivirus (NC), and small interfering RHOA (si-RHOA) were designed by Shanghai Jikai Biological Co., Ltd. All process reference reagent manufacturers’ instructions for transfection of PC cells PANC-1 and BXPC-3.

### qRT-PCR

TRIzol was used to extract RNA from PC tissues and cells following the company instructions. RNA purity and concentration were determined using a UV spectrophotometer. RNA was reverse transcribed into cDNA using the RT-PCR kit (Vazyme, Shanghai, China) of the Super Script III First-Strand Synthesis System. The relative amount of target genes was determined by normalization with the internal control (GAPDH mRNA for mRNA, U6 for miRNA). The sequences of all primers used in this study are shown in Supplementary Table 1.

### Nuclear-cytoplasmic RNA isolation

Nuclear and cytoplasmic RNA in PC cells were collected using the Nuclear/Cytosol Fractionation Kit (Biovision, USA) in accordance with the manufacturer’s instructions. RNA was evaluated by qRT-PCR. U6 and GAPDH mRNA were used as nuclear and cytoplasmic RNA internal controls.

### FISH

The LINC00857 probe was purchased from Ribobio (Guangzhou, China). PANC-1 and BXPC-3 cells were cultured and immobilized in 4% paraformaldehyde for 30 min at 37 °C. After washing in PBS and permeabilizing by 0.1% Triton-X-100 for 10 min, the cells were incubated with probes for 2 h. DAPI was used to label cell nuclei for 5 min and the images were captured using a confocal microscope.

### CCK-8 assay

Cells were plated into 96-well plates (2 × 10^3^ cells/well). After various times points (6, 24, 48, or 72 h), CCK-8 reagent (Boster, Wuhan, China) was added and samples were incubated for 2 h. Absorbance was read at 450 nm using a microplate reader.

### Colony-formation assay

Cells were cultured in a six-well plate (1 × 10^3^ cells/well). After two weeks, 4% paraformaldehyde was used to fix cells for 15 min, and then cells were stained with crystal violet for 20 min. After washing cells with PBS, the number of colonies was counted.

### 5-Ethynyl-2′-deoxyuridine (EdU) assay

EdU reagent was purchased from Ribobio (Guangzhou, China). Transfected cells were cultured in confocal dishes and then washed and fixed after 24 h. Next, 0.2% Triton X-100 (Boster, Wuhan, China) was used to treat cells for 10 min. Cells were then incubated with the EdU reagent for 25 min and stained with Hochest33258 for 10 min. Images were captured by a fluorescence microscope.

### Migration and invasion assays

For the wound-healing assay, cells of different treatment groups were plated in wells of a six-well plate. After cells achieved monolayer growth, a 200 μl pipette tip was used to create a scratch in the cell layer, and then cells were cultured in serum-free complete medium. Images were obtained using a light microscope at 0 h and 24 h.

For Transwell assays, transfected PC cells (2 × 10^4^ per well) in serum-free medium were seeded in the top chambers of Transwell systems with or without Matrigel (BD Biosciences, USA) in 24-well plates. The bottom chambers contained 10% FBS-containing complete medium. After culturing for 24 h, cotton balls were used to remove unattached cells in the upper chamber; the insert was fixed with paraformaldehyde and stained with crystal violet. Cells were imaged using a light microscope.

### Western blot

PC cells were harvested by cell scrapers and then subjected to ultrasonic disruption and centrifugation. BCA protein quantification method was used to determine the protein concentration and an appropriate amount of 5× loading buffer was added to each sample. Denatured samples were separated on 10% SDS-PAGE gels and transferred to a membrane. The membrane was blocked in 5% milk, washed in Tris-buffered saline (TBST), and incubated with the following primary antibodies for 12 h for 4 °C: E-cadherin (1:500) (Abcam, USA), Vimentin (1:500) (ABclonal), N-cadherin (1:500) (ABclonal), RHOA (1:1000) (ABclonal), ROCK1 (1:1000) (ABclonal), GAPDH (1:3000) (ABclonal). After three washes in PBS, secondary antibodies were added for 2 h. ECL luminescence reagent was added to visualize the bands, and GAPDH was set as an internal control. This experiment was repeated three times independently.

### Animal experiments

BALB/c nude mice (5-week-old females) were purchased from Zhejiang Weitong Lihua Laboratory Animal Technology Co., Ltd. Stably transfected PANC-1 cells (sh-NC, sh-LINC00857#1, or sh-LINC00857#2) (3 × 10^6^ cells suspended in 150 μl PBS) were injected into the skin of the left leg of nude mice (*N* = 4 for each group). Tumor volume was measured once a week and the mice were sacrificed 6 weeks later. The tumors were removed, weighed, and stored in paraformaldehyde for further study.

We constructed a liver metastasis model by injecting stably transfected PANC-1 cells (3 × 10^6^ cells in 100 μl PBS) into the tail vein of mice. After 10 weeks of feeding, the mice were sacrificed and the livers in mice were removed for analysis of tumor foci on the liver.

All animal procedures were approved by the Ethics Committee of Guizhou Medical University and followed the legal mandates and national guidelines for the care and maintenance of laboratory animals.

### Immunohistochemical staining

Mouse tumor specimens were embedded in paraffin. The tissue sections were incubated with primary antibodies against PCNA and Ki-67, followed by a secondary antibody labeled with biotin. Samples were analyzed under a fluorescence microscope.

### RNA pull-down assay

Control probes and biotin-labeled LINC00857 probes were synthesized by Genechem (Guangzhou, China). C-1 magnetic beads (Life Technologies Corporation, USA) were incubated with probes for 2 h at room temperature. The transfected PC cells were lysed on ice and incubated overnight with the bead-probe mixture at 4 °C. Finally, the samples were collected and purified using the RNeasy mini-kit (Qiagen, USA). LINC00857 expression abundance in the RNA complex was examined by qRT-PCR.

### Luciferase reporter assays

PANC-1 and BXPC-3 cells were plated in 24-well plates. miR-130b mimics or corresponding NC mimics were co-transfected along with reporter vectors in cells. After 48 h, the cells were lysed, and firefly and Renilla luciferase activities were detected. The firefly luciferase activity was used as a control to quantify the relative activity.

### Statistical analysis

All data were analyzed using SPSS software. The *t* test was used to determine statistical variations between two groups, and more than two groups were compared using one-way analysis of variance analysis. The co-expression relationship between LINC00857, miR-130b, and RHOA was analyzed by the Pearson correlation test. The relationship between LINC00857 and clinical traits of patients with PC was analyzed by Fisher’s exact test. A two-tailed *P* value of < 0.05 was regarded as statistically significant.

## Supplementary information

supplementary material

supplementary material

Supplement figure 1

supplementary material

supplementary material

supplementary material

supplementary material

## Data Availability

All data generated and analyzed during this study are included in this published article and available on request.
