# Correction: Long noncoding RNA LINC00857 promotes pancreatic cancer proliferation and metastasis by regulating the miR-130b/RHOA axis

**DOI:** 10.1038/s41420-025-02871-5

**Published:** 2026-01-28

**Authors:** Peng Chen, Zhirui Zeng, Jie Wang, Wenpeng Cao, Chunzhuo Song, Shan Lei, Yichuan Li, Zhangxia Ren

**Affiliations:** 1 Department of General Surgery, Guang’an People’s Hospital, Guang’an, Sichuan China; 2 Basic Medical College of Guizhou Medical University, Guiyang, Guizhou China; 3 Department of Hepatobiliary Surgery, Renmin Hospital of Wuhan University, Wuhan, Hubei China; 4 Department of Hepatobiliary Surgery, The Second affiliated Hospital of Army Medical University, Chongqing, China

**Keywords:** Metastasis, Cancer microenvironment

Correction to: *Cell Death Discovery* 10.1038/s41420-022-01008-2, published online 13 April 2022

During the process of capturing Transwell images, I used very simple file names such as “sh” and “sh+inhibitor.” Unfortunately, this led to some images from the two projects being intermingled due to the file naming and sorting sequence.

In both studies, knockdown of the respective lincRNA reduced the invasive ability of pancreatic cancer cells, and this reduction was reversed upon treatment with the downstream microRNA inhibitor. As the trends were highly consistent between the two studies, the inadvertent mix‑up of images did not affect the overall conclusions of the work.

However, while organizing data for my subsequent manuscript, I became concerned that this earlier mix‑up could lead to potential issues once the new paper is published, as the two manuscripts might then share incorrectly used images. Therefore, I feel it is necessary to correct the affected figures based on the original image capture dates.

**Fig 2G-invasion-BXPC-3-sh-LINC00857#2-origin** 
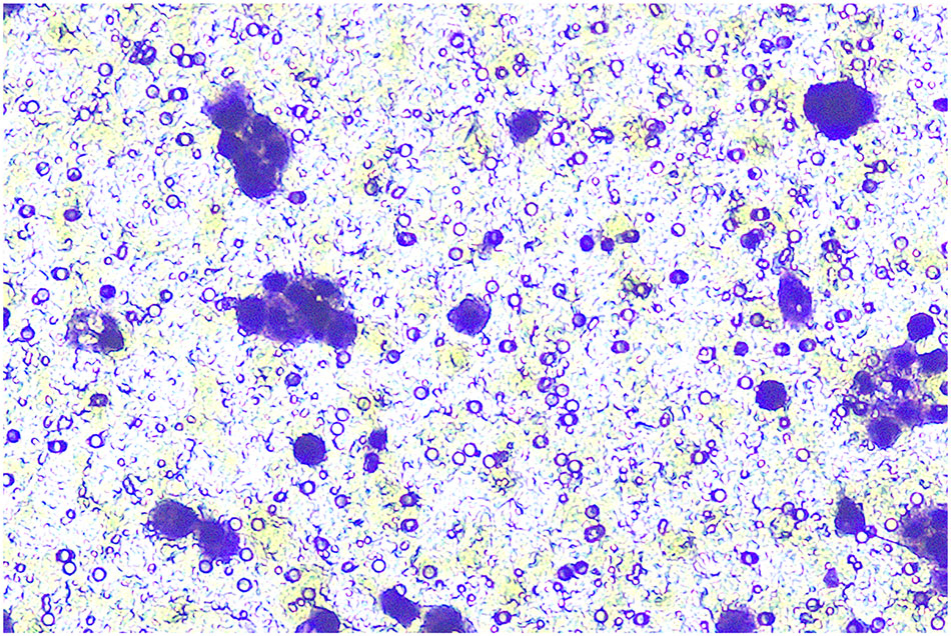


**Fig 2G-invasion-BXPC-3-sh-LINC00857#2-amend** 
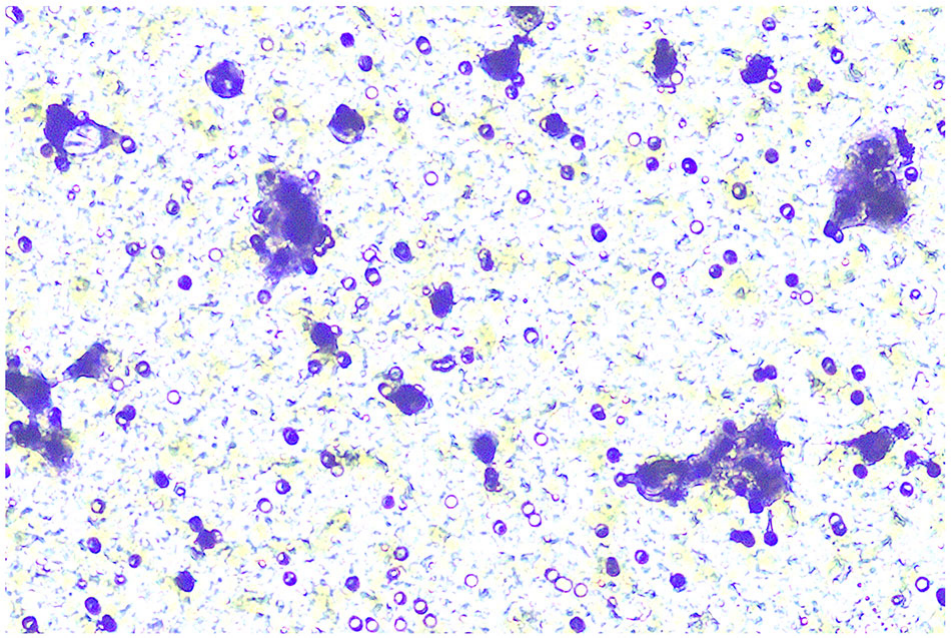


**Fig 2G-invasion-BXPC-3-sh-NC-origin** 
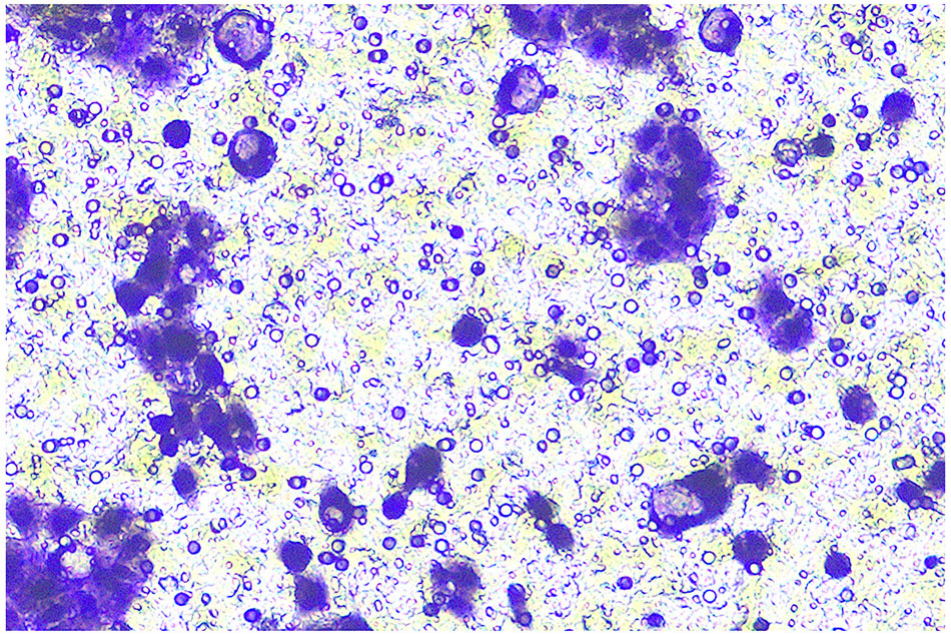


**Fig 2G-invasion-BXPC-3-sh-NC-amend** 
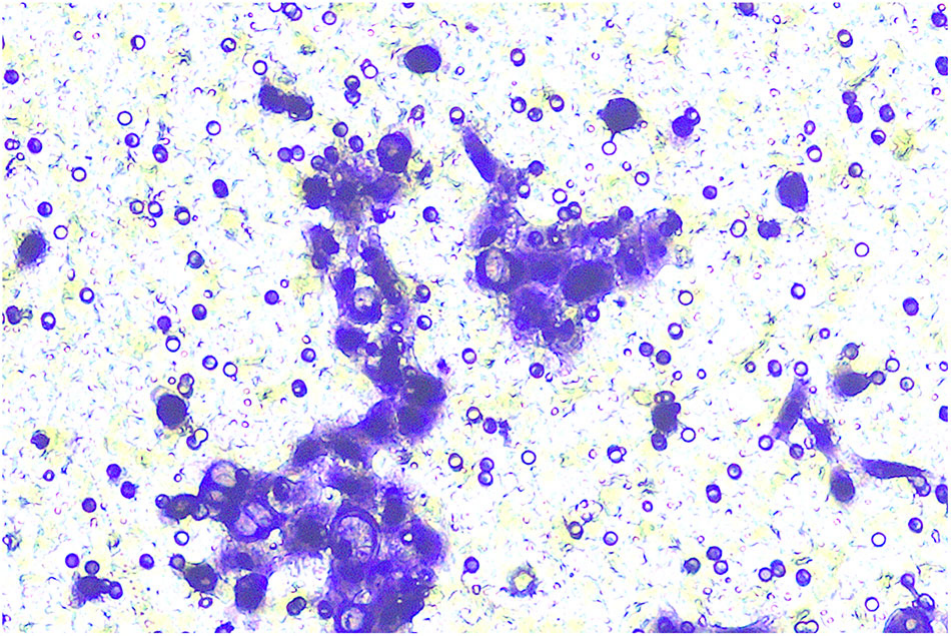


**Fig 5H-invasion-PANC-1-sh-LINC00857+inhibiters-origin** 
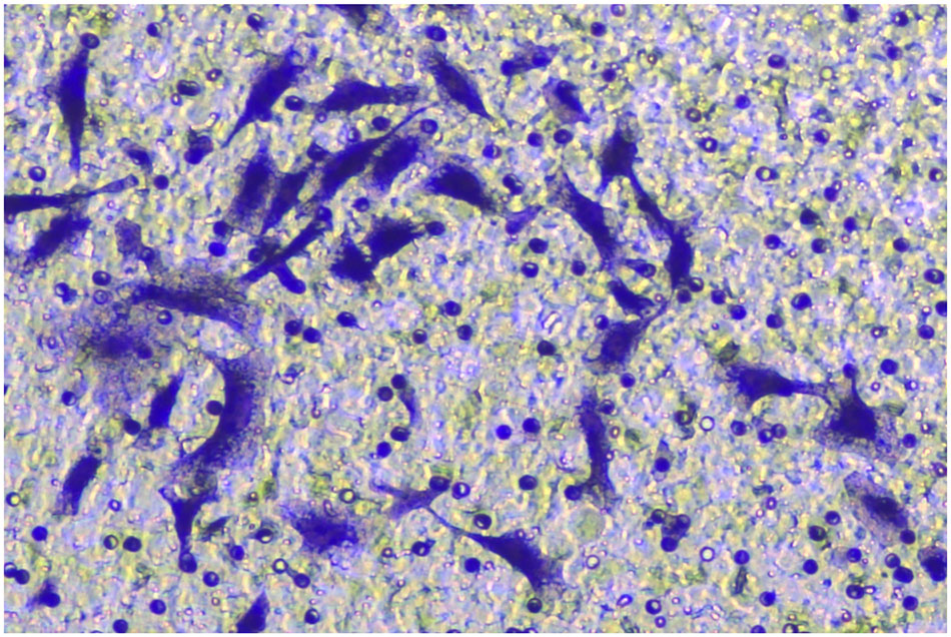


**Fig 5H-invasion-PANC-1-sh-LINC00857+inhibiters-amend** 
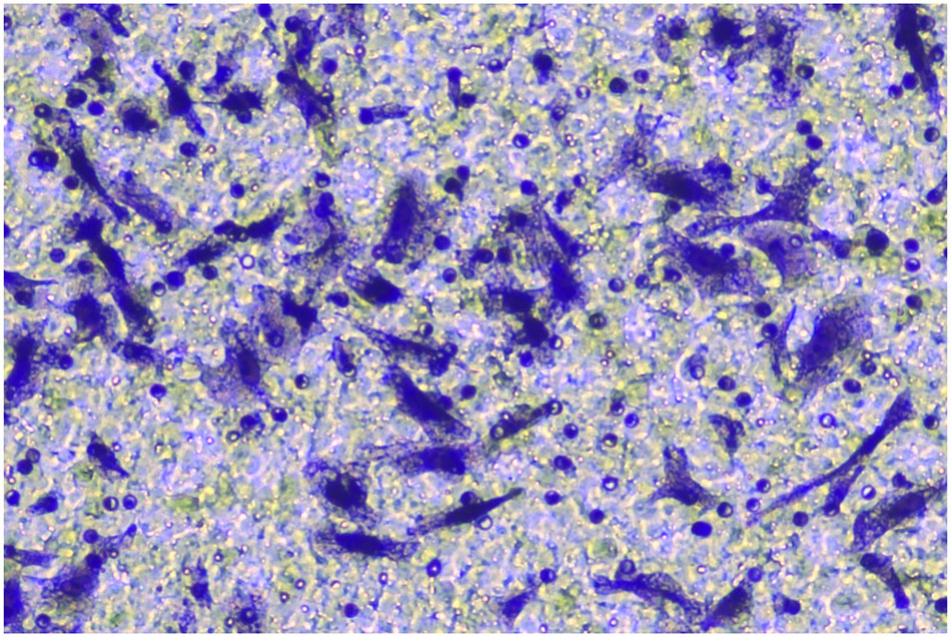


**Fig 5H-migration-PANC-1-sh-LINC00857+inhibiter-origin** 
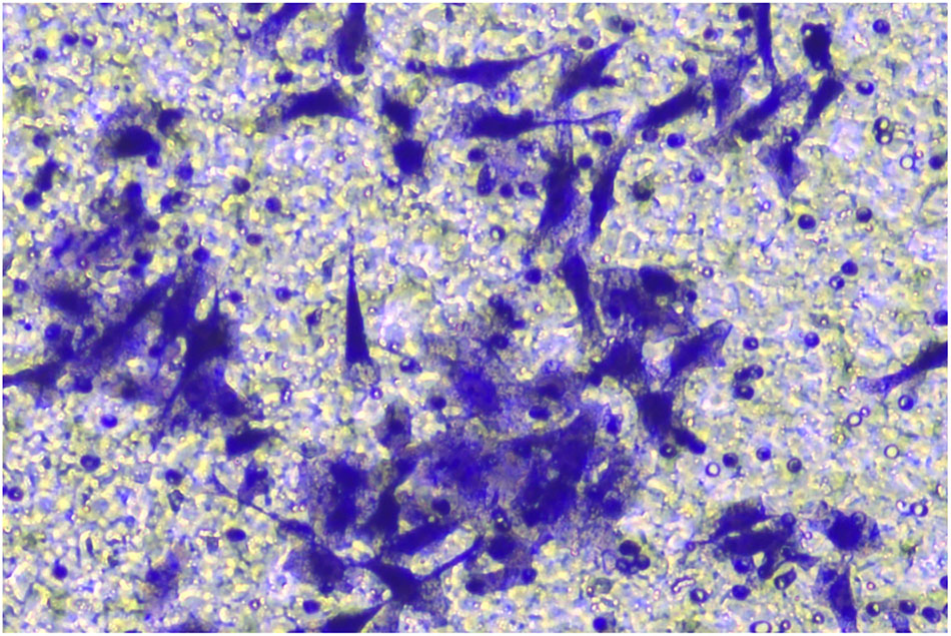


**Fig 5H-migration-PANC-1-sh-LINC00857+inhibiters-amend** 
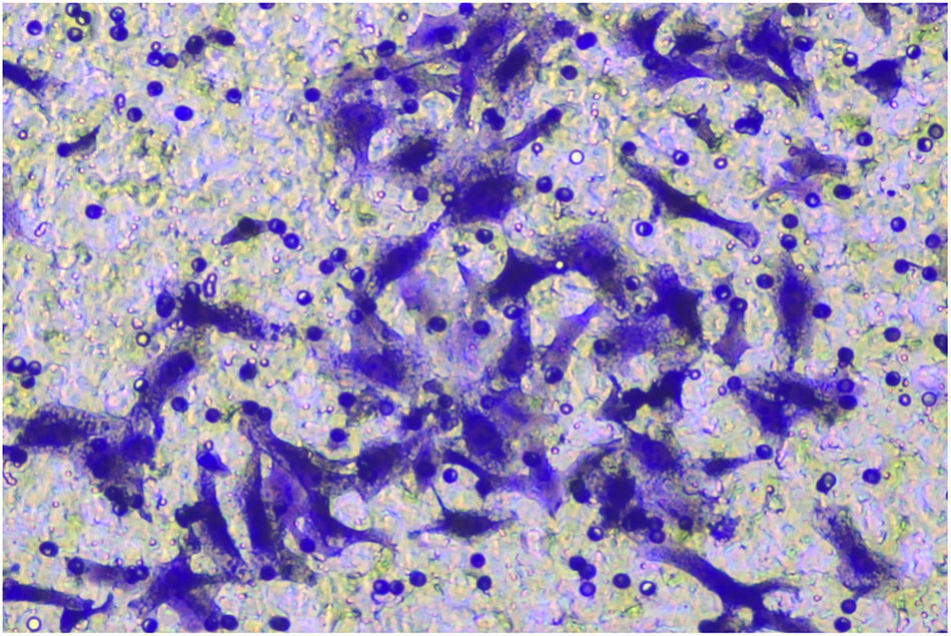


**Fig 5H-migration-PANC-1-sh-LINC00857-origin** 
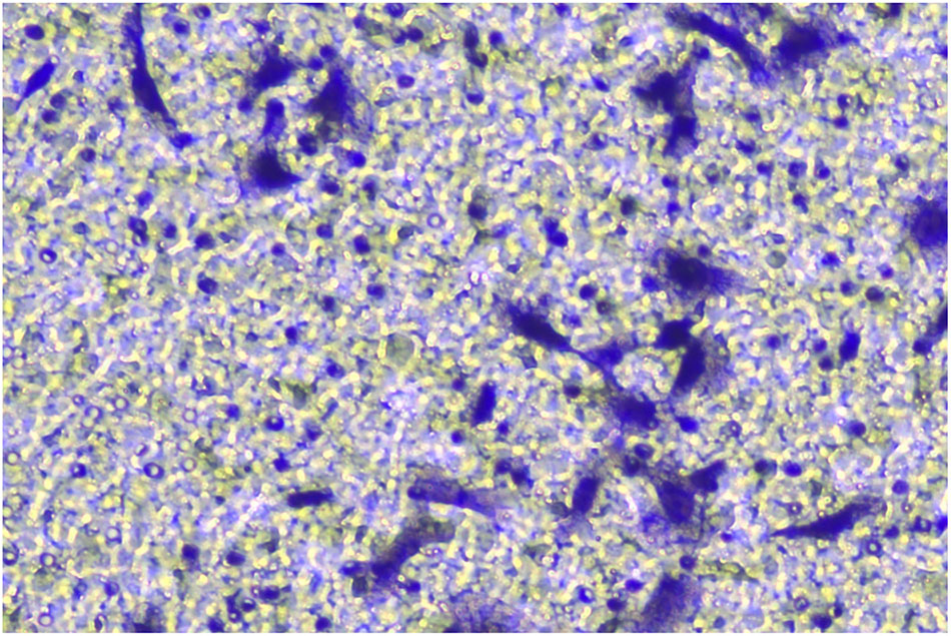


**Fig 5H-migration-PANC-1-sh-LINC00857-amend** 
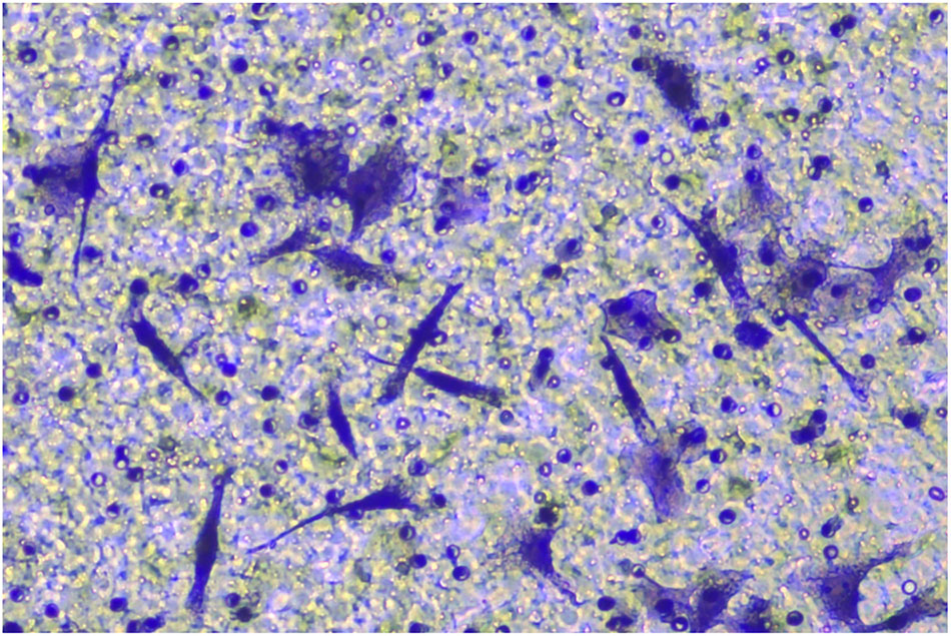


**Fig 8G-invasion-BXPC-3-NC-origin** 
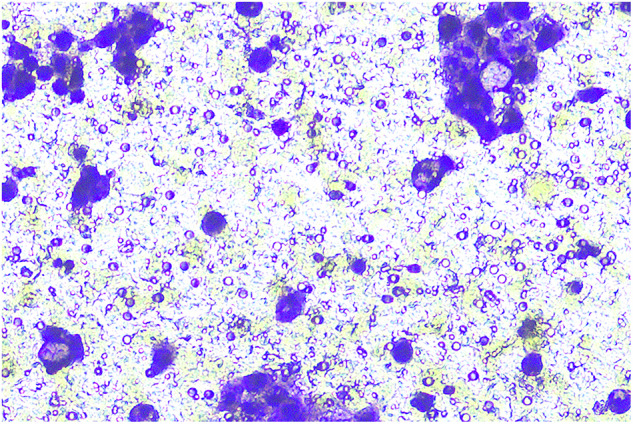


**Fig 8G-invasion-BXPC-3-NC-amend** 
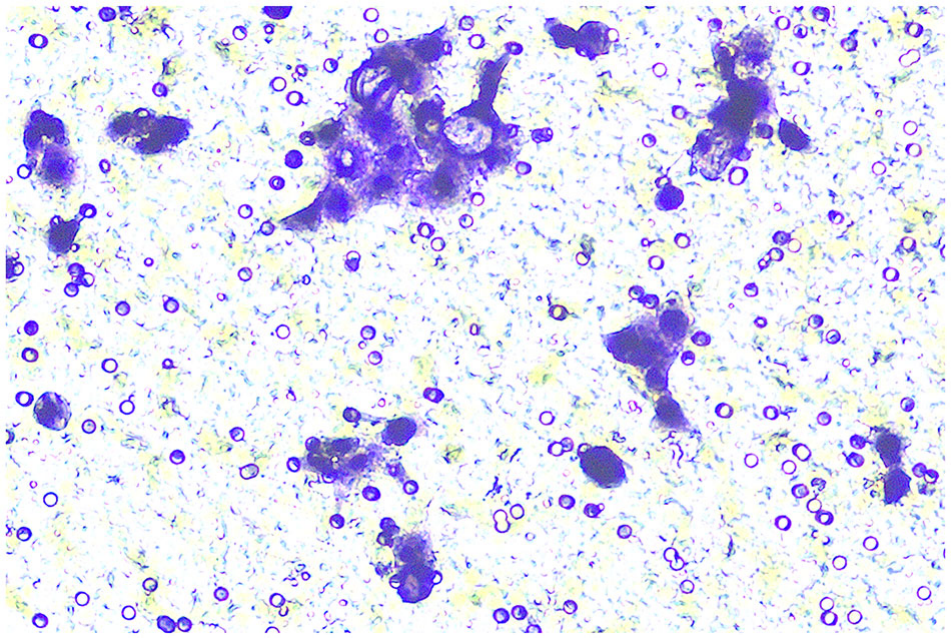


The original article has been corrected.

