# Supplementary material for: Long noncoding RNA LINC00857 promotes pancreatic cancer proliferation and metastasis by regulating the miR-130b/RHOA axis
Source: Cell Death Discov. 2022 Apr 13;8:198. doi: 10.1038/s41420-022-01008-2 (PMC9008000; doi:10.1038/s41420-022-01008-2)
Supplement: Supplementary file 1 — supplementary material [file 41420_2022_1008_MOESM1_ESM.doc]

The sequence of all primers

Gene name the sequence of primer(5’→3’)

LINC00857 Forward 5’- CCCCTGCTTCATTGTTTCCC-3’

Reverse 5’-AGCTTGTCCTTCTTGGGTACT-3’

miR-130b 5’-ACACTCCAGCTGGGCAGTGCAATGATGAAA-3’

miR-148b 5’-ACACTCCAGCTGGGTCAGTGCATC-3’

miR-106b 5’-CAAAGTGCTAACAGTGCAGGTAG-3’

RHOA Forward 5’-GGAAAGCAGGTAGAGTTGGCT-3’

Reverse 5’-GGCTGTCGATGGAAAAACACAT-3’

U6 forward 5’-CTCGCTTCGGCAGCACA-3’

Reverse 5’-AACGCTTCACGAATTTGCGT-3’

GAPDH forward 5’-CAATGACCCCTTCATTGACC-3’

Reverse 5’-TTGATTTTGGAGGGATCTCG-3’
