# Supplementary material for: Long noncoding RNA LINC00857 promotes pancreatic cancer proliferation and metastasis by regulating the miR-130b/RHOA axis
Source: Cell Death Discov. 2022 Apr 13;8:198. doi: 10.1038/s41420-022-01008-2 (PMC9008000; doi:10.1038/s41420-022-01008-2)
Supplement: Supplementary file 2 — supplementary material [file 41420_2022_1008_MOESM2_ESM.docx]

**Table 1. General clinicopathological characteristics of patients**

| **Clinical Epidemiology and Clinicopathologic Feature** |  | **LINC00857** | | ***p value*** |
| --- | --- | --- | --- | --- |
|  | **N** | **low expression** | **high expression** |  |
| All cases | 40 | 20 | 20 |  |
| **Age** |  |  |  |  |
| ≤60 | 17 | 7 | 10 | 0.523 |
| ＞60 | 23 | 13 | 10 |  |
| **Gender** |  |  |  |  |
| male | 26 | 12 | 14 | 0.741 |
| female | 14 | 8 | 6 |  |
| **Diameter of tumor** |  |  |  |  |
| ≤3 | 21 | 14 | 5 | 0.010 |
| ＞3 | 19 | 6 | 15 |  |
| **Tumor differentiation** |  |  |  |  |
| Well/moderate | 18 | 12 | 6 | 0.111 |
| Poor | 22 | 8 | 14 |  |
| **Pathological T** |  |  |  |  |
| T1/T2 | 16 | 12 | 4 | 0.023 |
| T3/T4 | 24 | 8 | 16 |  |
| **Lymph node metastasis** |  |  |  |  |
| negative | 18 | 13 | 5 | 0.025 |
| positive | 22 | 7 | 15 |  |

Note: Low/high by the sample median used Fisher’s exact test.

**P* <0.05 was considered to be statistically significant.
