# Supplementary figures and images for: Long noncoding RNA LINC00857 promotes pancreatic cancer proliferation and metastasis by regulating the miR-130b/RHOA axis
Source: Cell Death Discov. 2022 Apr 13;8:198. doi: 10.1038/s41420-022-01008-2 (PMC9008000; doi:10.1038/s41420-022-01008-2)

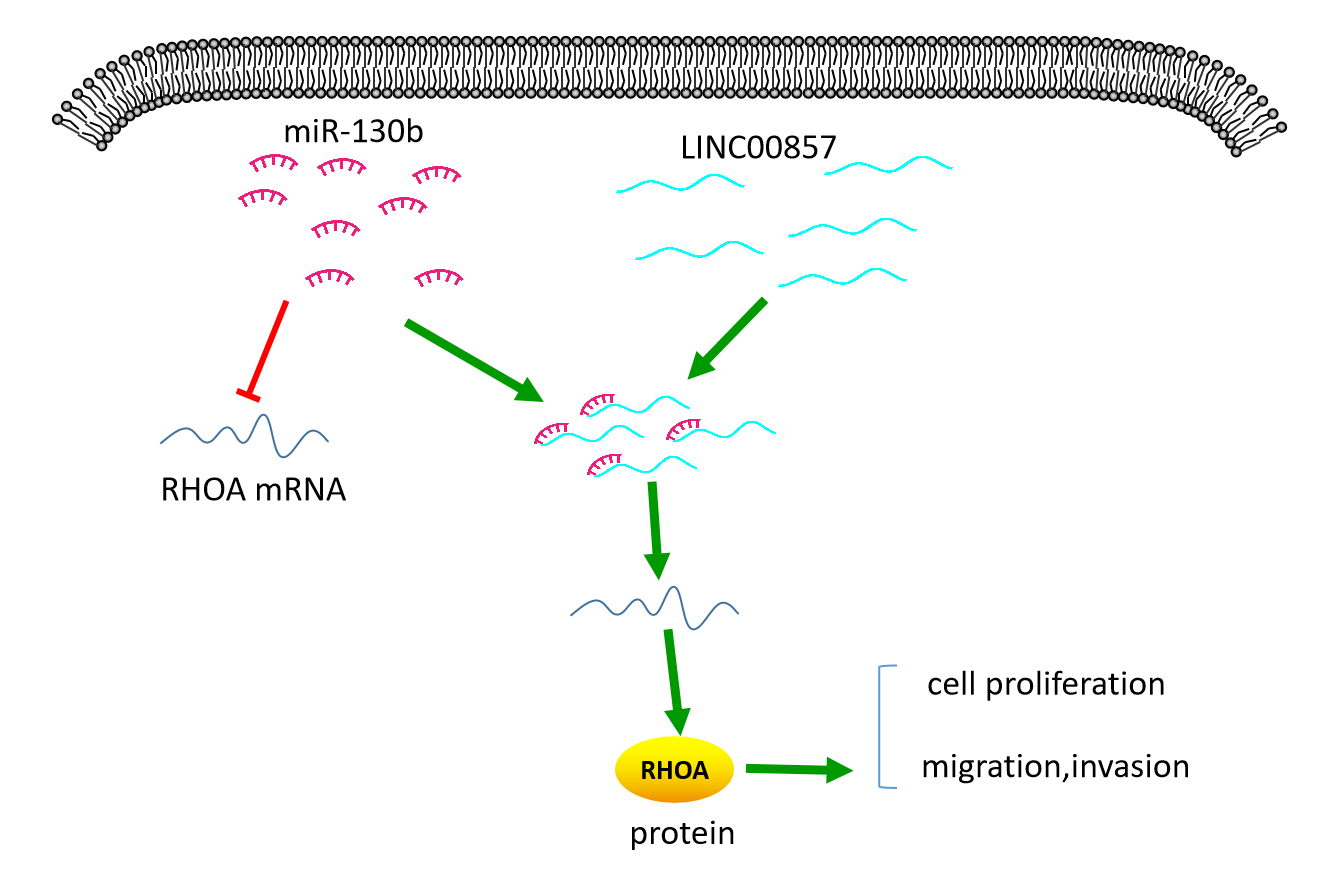

Supplement: Supplementary file 3 — Supplement figure 1 [file 41420_2022_1008_MOESM3_ESM.tif]

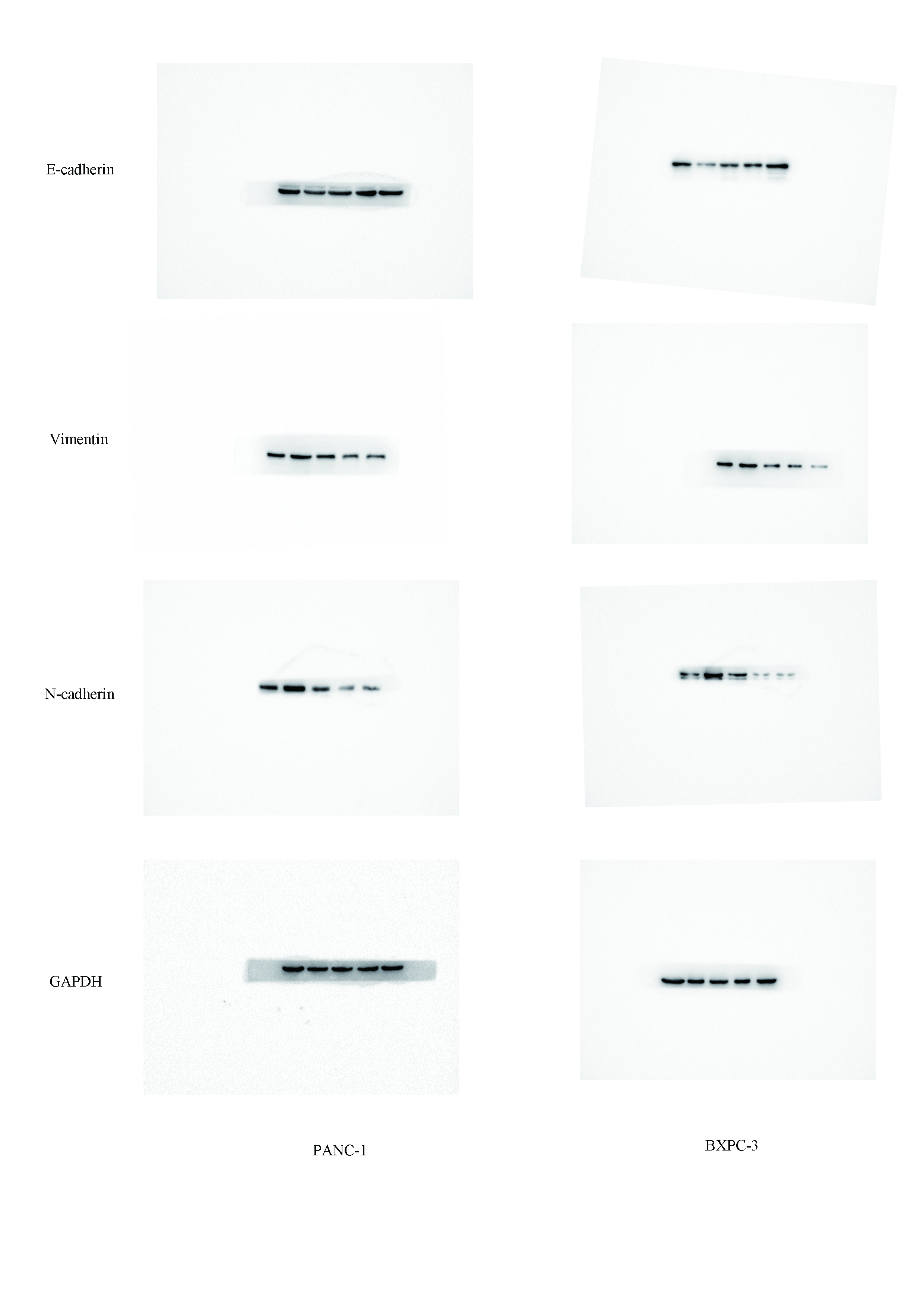

Supplement: Supplementary file 4 — supplementary material [file 41420_2022_1008_MOESM4_ESM.tif]

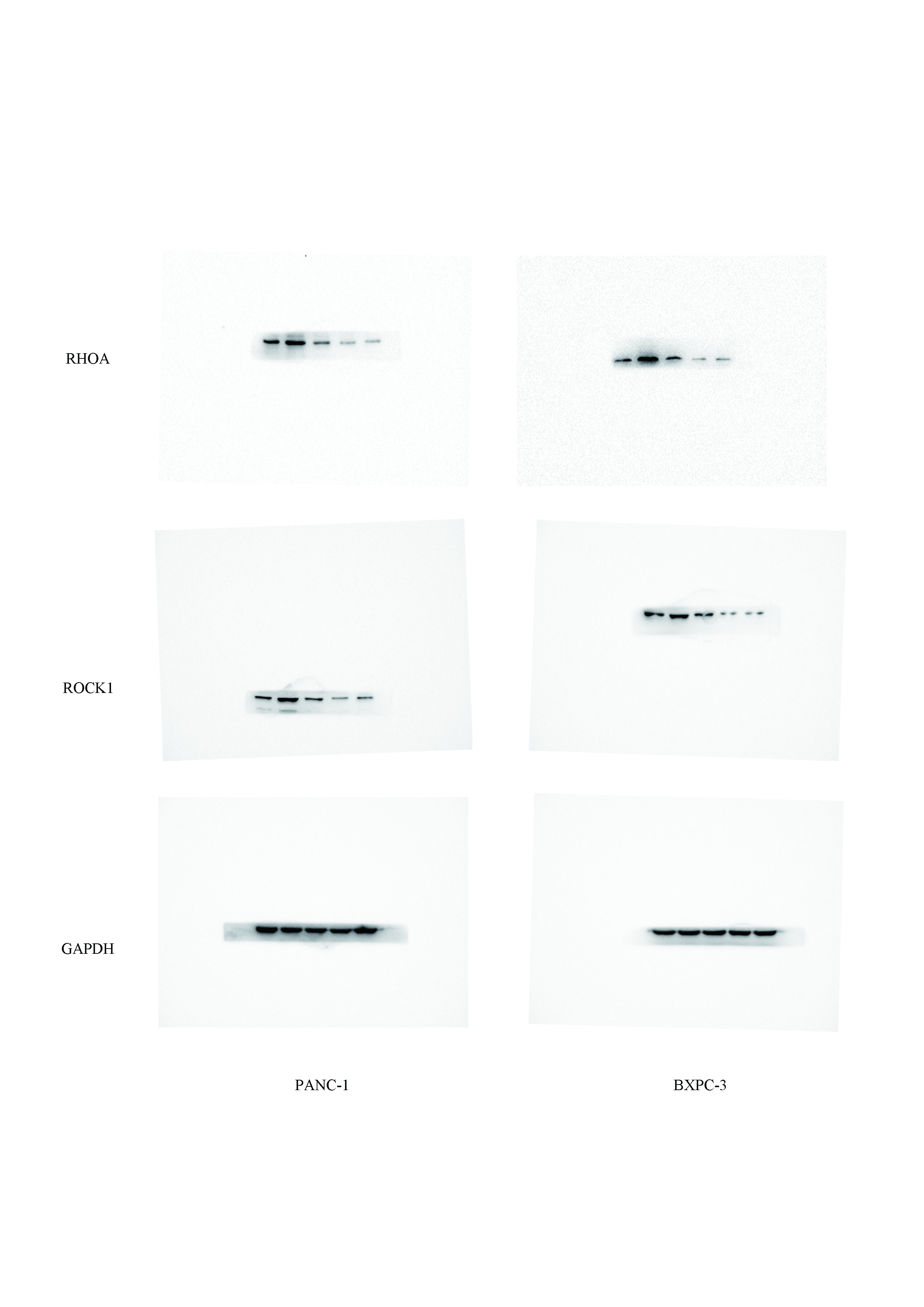

Supplement: Supplementary file 5 — supplementary material [file 41420_2022_1008_MOESM5_ESM.tif]

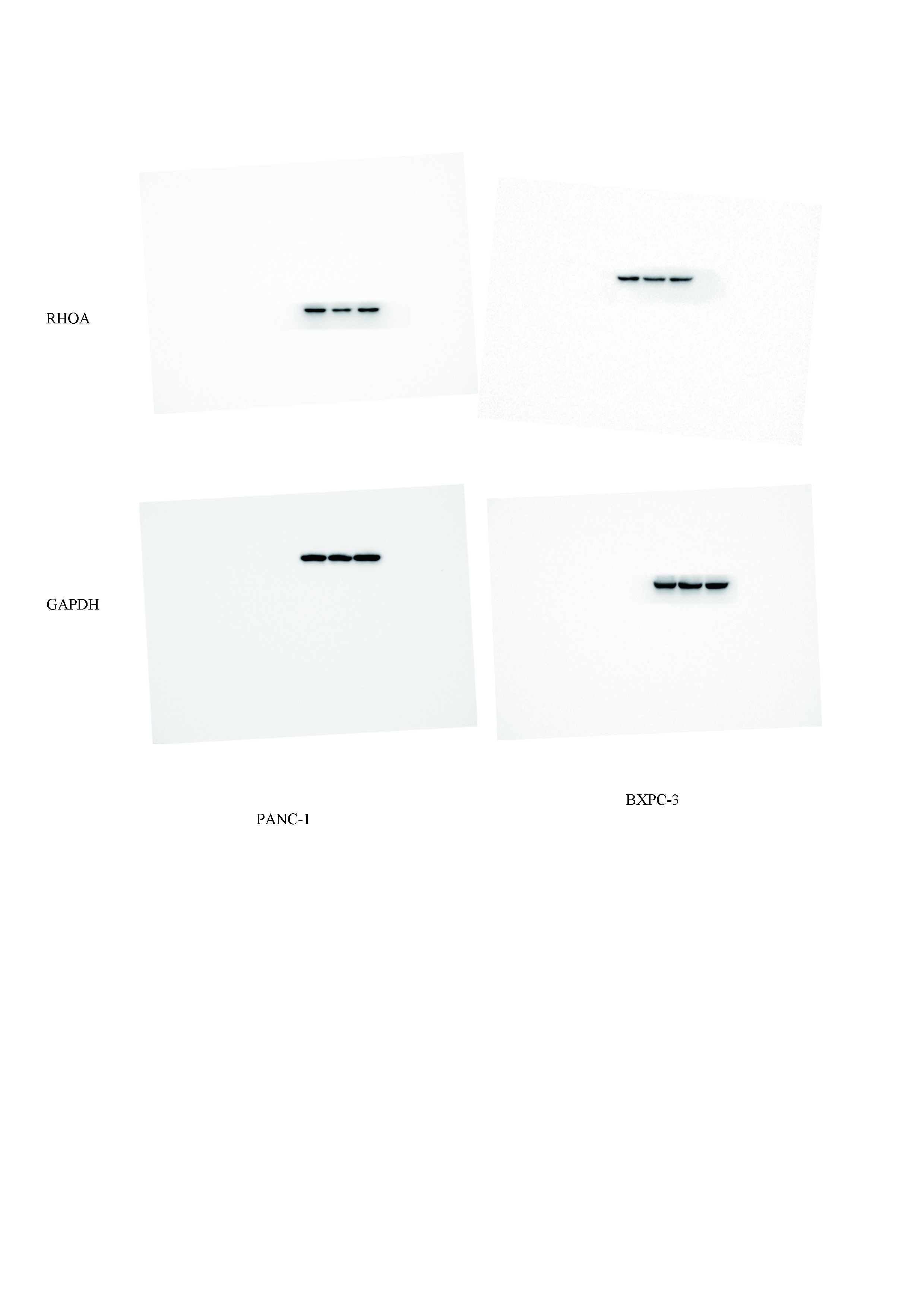

Supplement: Supplementary file 6 — supplementary material [file 41420_2022_1008_MOESM6_ESM.tif]

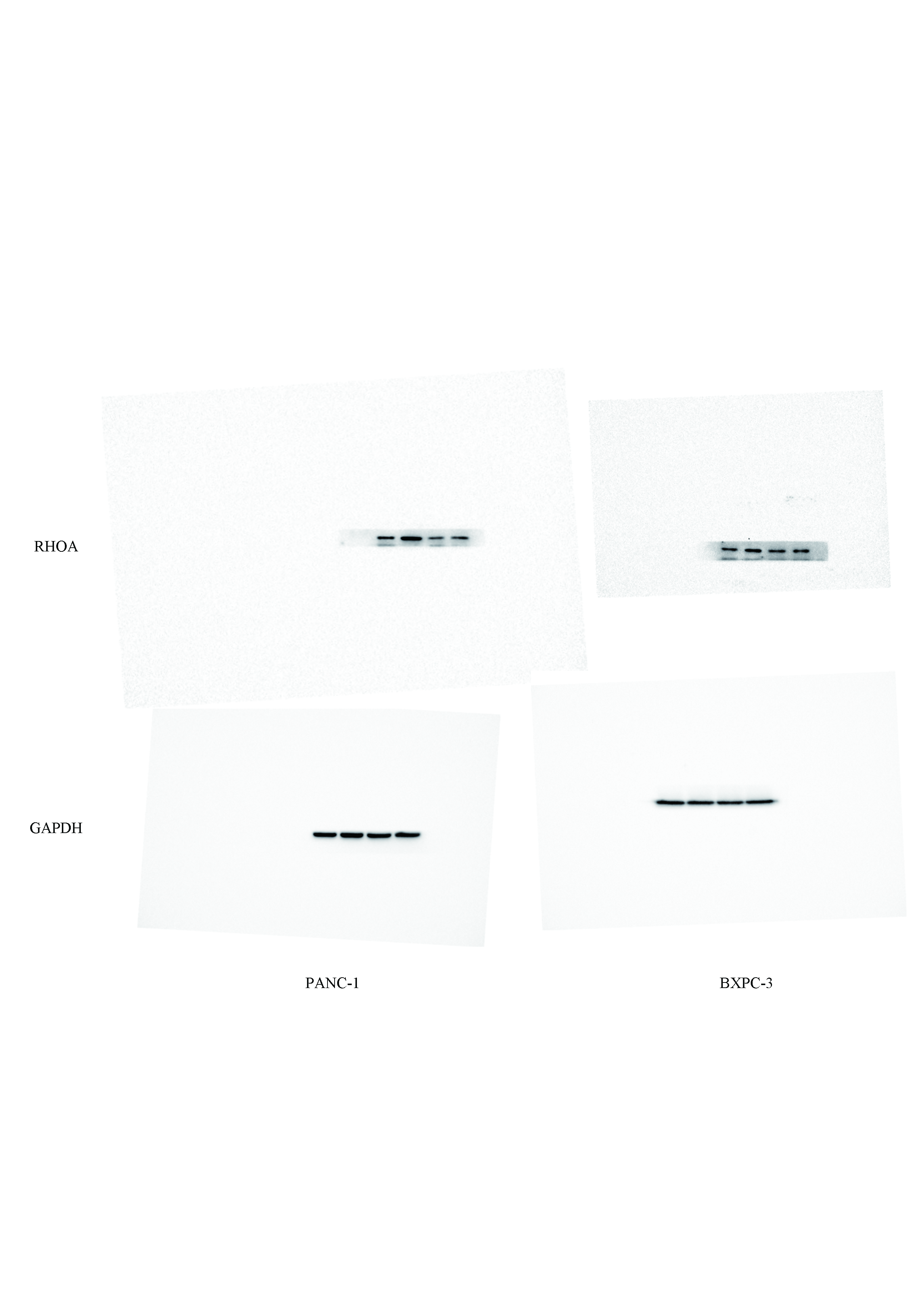

Supplement: Supplementary file 7 — supplementary material [file 41420_2022_1008_MOESM7_ESM.tif]
